# Concurrent validity of skin carotenoid status as a concentration biomarker of vegetable and fruit intake compared to multiple 24-h recalls and plasma carotenoid concentrations across one year: a cohort study

**DOI:** 10.1186/s12937-019-0500-0

**Published:** 2019-11-21

**Authors:** Lisa Jahns, LuAnn K. Johnson, Zach Conrad, Michael Bukowski, Susan K. Raatz, Stephanie Jilcott Pitts, Youfa Wang, Igor V. Ermakov, Werner Gellermann

**Affiliations:** 1 United States Department of Agriculture, Agricultural Research Service, Grand Forks Human Nutrition Research Center, 2420 2nd Avenue North, Grand Forks, ND 58203 USA; 2 Department of Health Sciences, William & Mary, 251 Ukrop Way, Williamsburg, VA 23185 USA; 3 Department of Food Science and Nutrition, 225 Food Science and Nutrition, 1334 Eckles Ave, St. Paul, MN 55108 USA; 4 Department of Public Health, Brody School of Medicine, East Carolina University, 115 Heart Drive MS 660, Greenville, NC 27834 USA; 5 Department of Nutrition and Health Sciences, College of Health, Ball State University, Muncie, IN 47306 USA; 6 Longevity Link Corporation, University of Utah Research Park, 391 Chipeta Way Suite E, Salt Lake City, UT 84108 USA

**Keywords:** Resonance Raman spectroscopy, Reflection spectroscopy, Skin carotenoids, Biomarkers, Vegetables, Fruits, ASA24 Dietary Assessment Tool, Dietary intake, Women, Cohort study

## Abstract

**Background:**

Biological markers of vegetable and fruit (VF) intake are needed both for nutrition surveillance and for the evaluation of nutrition interventions. Optically assessed skin carotenoid status (SCS) has been proposed as a marker of intake but there are few published validity studies to date. Therefore, the objective of the study was to examine the concurrent validity of multiple methods of assessing VF intake cross-sectionally and seasonally over one year and to discuss the relative merits and limitations of each method.

**Methods:**

Fifty-two 40–60 y old women completed a 1-year longitudinal study that included 1) SCS assessment using resonance Raman spectroscopy (RRS) and using pressure-mediated reflection spectroscopy (RS) at 12 timepoints, 2) thirty-six 24-h recalls using the Automated Self-Administered 24-Hour Dietary Assessment Tool (ASA24; total 1866 recalls), and 3) plasma carotenoid concentrations measured every 3 months. Pearson correlation coefficients and mixed linear models were used to estimate pairwise correlations between RRS, RS, ASA24, and plasma carotenoids.

**Results:**

RS and RRS were strongly correlated at baseline and over the year (*r* = 0.86 and 0.76; respectively, *P* < 0.001). RS was strongly correlated with plasma carotenoids at baseline (*r* = 0.70) and moderately across the year (*r* = 0.65), as was RRS (*r* = 0.77 and 0.69, respectively, all *P* < 0.001). At baseline, self-reported VF was weakly correlated with RRS (*r* = 0.33; *P* = 0.016), but not with RS or plasma carotenoids. Across the year, self-reported VF intake was weakly correlated with both RS (*r* = 0.37; *P* = 0.008), RRS (*r* = 0.37; *P* = 0.007), and with plasma carotenoids (*r* = 0.36; *P* < 0.008).

**Conclusions:**

SCS as measured by RS and RRS is moderately to strongly correlated with plasma carotenoid concentrations both cross-sectionally and longitudinally, indicating that it can be a powerful tool to assess carotenoid-rich VF intake in populations.

**Clinical trial registry:**

This trial was registered at ClinicalTrials.gov as NCT01674296.

## Background

Adequate vegetable and fruit (VF) intake is associated with reduced risk of several chronic diseases [1]. Yet in many populations intake is either declining [2] or remaining stable at insufficient amounts [3]. Despite large investments in initiatives like the 5-A-Day program [4, 5], increasing VF intake still confounds clinicians and public health nutrition professionals. Accurately measuring VF intake is crucial for population surveillance and for evaluating the efficacy of interventions, but a key dilemma in this pursuit has been inherent bias and error in self-reported measurement tools such as food frequency questionnaires, food records, and 24-h recalls [6].

There is a need for biological markers of dietary intake [7]. Ideally, a biomarker would pass through the body unaffected and be recovered in its entirety. To date, no such recovery biomarker exists for VF, but concentration biomarkers have been identified. Concentration biomarkers reflect the status of the marker of interest in the body and therefore are affected by individual differences in absorption, transport, and metabolism [7]. The Institute of Medicine called blood carotenoid concentrations the best biomarker of VF intake [8]; however, collecting and analyzing blood carotenoids is prohibitive for many studies. Optically-assessed skin carotenoid status (SCS) has been proposed as a concentration biomarker of carotenoid intake for monitoring VF intake and for assessing change in VF intake as a result of interventions [9]. SCS has favorably compared to carotenoid content of biopsied skin [10, 11] and to blood carotenoid concentrations [10, 12]. SCS was also responsive to changes in VF intake in a controlled feeding study [13]. Seasonal differences in sun exposure and VF intake are purported to affect SCS, but the literature is mixed [13, 14].

There are two methodologies for measuring SCS; resonance Raman spectroscopy (RRS) [15, 16] and reflection spectroscopy (RS) [9, 16, 17]. RRS has been used in most of the existing studies and found to be representative of longer-term intake than blood carotenoid concentrations, perhaps by 1–2 weeks [10, 15, 18, 19]. RS represents an improvement over RRS as it corrects for effects of residual chromophores like blood and melanin in the skin [16]. RS was validated against plasma carotenoids in a racially/ethnically diverse sample [20] and is correlated with RRS [16]; yet, to the best of our knowledge, no studies have cross-validated multiple objective measures of VF intake (RRS, RS, and plasma carotenoid concentrations) and subjective measures of VF intake (self-report). This represents an important research gap that can be used to improve assessment of VF intake and change data.

Therefore, the purpose of this study was to examine the concurrent validity of multiple methods of assessing VF intake. We estimated baseline and yearlong pairwise correlations between 4 methods of assessing VF intake: 1) plasma carotenoid concentration, 2) multiple 24-h recalls, 3) RRS, and 4) RS. A secondary aim of the study was to examine potential seasonal differences in the measurements obtained using each method.

## Subjects and methods

### Study design and subjects

The present study reports a secondary outcome using data from an existing study and should be considered exploratory. The study sample consisted of 52 mid-life women who completed the Life in all Seasons (LENAS) study. LENAS utilized a closed cohort design and was conducted in two groups between July 2012 and July 2014. The study was designed to measure seasonal changes in diet, physical activity, and body composition over a 1-year period. Details have been reported elsewhere [21, 22]. Seventy-three women attended informational meetings and 61 signed informed consents. Fifty-four women began the study and two dropped out due to time constraints. Briefly, over a 1-year period, women completed 24-h diet recalls (24-HR) every 10 days at home, had their SCS measured 12 times (monthly), and had plasma collected 4 times (once every three months at the midpoint of each season). Individuals were recruited by advertisements posted at the University of North Dakota and the surrounding area. Inclusion criteria were ages 40–60 years, body mass index (BMI; kg/m^2^) between 18 and 35, access to high-speed internet, and weight being stable for the previous 6 months (no more than ± 4.5 kg). The majority (96%) of the participants were non-Hispanic white, with a mean age of 49.4 ± 0.8 years and mean BMI of 26.5 ± 0.6; 33% were affected by overweight and 23% by obesity [21]. None of the participants reported current smoking. The study protocol was approved by the Institutional Review Board of the University of North Dakota and all participants provided informed written consent.

### Skin carotenoid measurements

Skin carotenoid measurements were taken by both RRS and RS twelve times over the year, approximately one month apart. The two devices used were built by the authors (IVE and WG) for use with nutrition studies. RRS uses a 488 nm solid-state laser for blue light excitation of the tissue carotenoids. The laser light is directed onto the skin via optical fiber delivery and a collection module is placed in contact with the palm of the hand. Light backscattered from the skin is routed via a second fiber to a spectrograph interfaced with a cooled charge coupled device detector array. The recorded spectrum is analyzed for resonance Raman response of the skin carotenoids at their carbon double bond (C=C) frequency at 1525 cm^− 1^, which appears at around 527 nm in the Raman spectrum. Since almost all carotenoid subspecies contribute almost equally to this frequency (excluding phytoene and phytofluene) [11], RRS intensity can be used as a measure for total carotenoid content. The instrument sensitivity was checked daily against a calibration standard (sodium nitrate), and individual skin carotenoid RRS intensities were calibration-adjusted. The intraindividual variability between the 3 scans is 5% on this instrument.

Instead of a laser, the RS methodology uses a broad-band white light to measure skin carotenoids and other chromophores like melanin and hemoglobin directly through their respective absorptions in the 400–750 nm spectral window [17]. The reflected light is routed to a spectrograph coupled with a room-temperature detector array. The subject applies gentle pressure against a lens to temporarily squeeze blood out of the tissue that reduces potentially confounding effects of chromophores such as hemoglobin. The data processing algorithm also adjusts for the potentially confounding effects of melanin and tissue scattering. In this study, a prototype instrument was used that took measurements in the thumb; current commercially available technology takes measurements in the fingers. The intra-individual variability of the prototype instrument varies between 0.5 and 14%, depending on the individual. Each individual’s palm (RRS) and thumb (RS), were scanned 3 times and the average value used in the analysis.

### Dietary intake of vegetables, fruits, and carotenoids

Approximately every 10 days, participants completed an online dietary recall using the US National Cancer Institute (NCI)‘s Automated Self-Administered 24-h Dietary Assessment Tool (ASA24–2011) [23, 24]. ASA24 prompts individuals to record everything consumed in the previous 24 h from midnight to midnight using detailed probes and has been validated against interviewer-administered 24-h recalls and actual intakes [25, 26]. Provided reports included servings of VF derived from the USDA Food and Nutrient Database for Dietary Studies 4.0 [27] and the USDA MyPyramid Equivalents Database 2.0 [28], and dietary carotenoids (mg) estimated using data from the USDA Standard Reference 22 [29] database. Participants completed a total of 1866 diet recalls with a 92% response rate for all 36 recalls (range 33–37); no recalls were excluded as day-to-day variation in intake was expected over the course of the year. Participants were not provided feedback or results from their recalls.

### Blood sample and carotenoid analysis

At the midpoint of each meteorological season [summer (July), fall (October), winter (January), and spring (April)], fasting blood samples were taken by a trained phlebotomist, centrifuged and immediately stored at − 80 °C. Human plasma carotenoid (α-, β- carotenes, β-cryptoxanthin, lycopene and lutein/zeaxanthin) analysis was performed using Bukowski, et al’s “dilute-and-shoot” [30] method by LC-MS/MS (LCMS-8050, Shimadzu). Under reduced lighting a 10 μL aliquot of human plasma was combined with 990 μL of methanol containing internal standard and was centrifuged to remove protein. The supernatant was transferred to 1-mL amber glass vial for analysis. A volume of 25 μL was injected onto a C-30 column (YMC Carotenoid, 10 × 2 mm, 3 μm particle size) with an initial 9:1 methanol water mobile phase, which concentrated analytes on the front of the column, allowing for the removal of phospholipid interferences that suppress ionization. After the washout period, the mobile phase transitioned over two minutes to 70:20:10 acetonitrile: methylene chloride: methanol (20 mM ammonium acetate, 0.3% acetic acid). Analytes were eluted over 35 min following the order observed by Melendez-Martinez [31] and were quantitated against the internal standard of tocol. This method was validated by analysis of all three levels of NIST SRM 968e. Measured values for all analytes were within the stated tolerance for this SRM.

As an additional validation step, a set of standard addition experiments were performed. Four aliquots of plasma were spiked with increasing levels of the analytes from a standard solution. The measured concentrations for these samples were plotted against the size of the spike added to the plasma and fit with a linear regression. The y-intercepts for these regressions agreed within 2–5% of the measured concentrations for the un-spiked plasma sample. This indicated that there were no matrix effects or artifacts from sample preparation.

### Statistical analysis

RRS, RS, and self-reported dietary VF and carotenoid values are presented as means ± standard error of the mean (SEM). Plasma carotenoid values were skewed according to the Kolmogorov-Smirnov test, so values were log-transformed for analysis. Back-transformed means -1SEM, +1SEM are presented.

Effects of season were tested using mixed linear models, in which season was the fixed effect and participant was the random effect. For RRS, RS and self-reported dietary VF and carotenoid values, multiple measurements obtained for each participant during each season were averaged and used as the dependent variable in the model. If the overall model was significant, Tukey’s contrasts were used to make pairwise comparisons among all seasons.

Pairwise correlations between baseline measures of carotenoid status and intake and VF intake obtained by the 4 methods (i.e. RRS, RS, plasma, and self-report) were estimated using Pearson correlations.

**Fig. 1:**
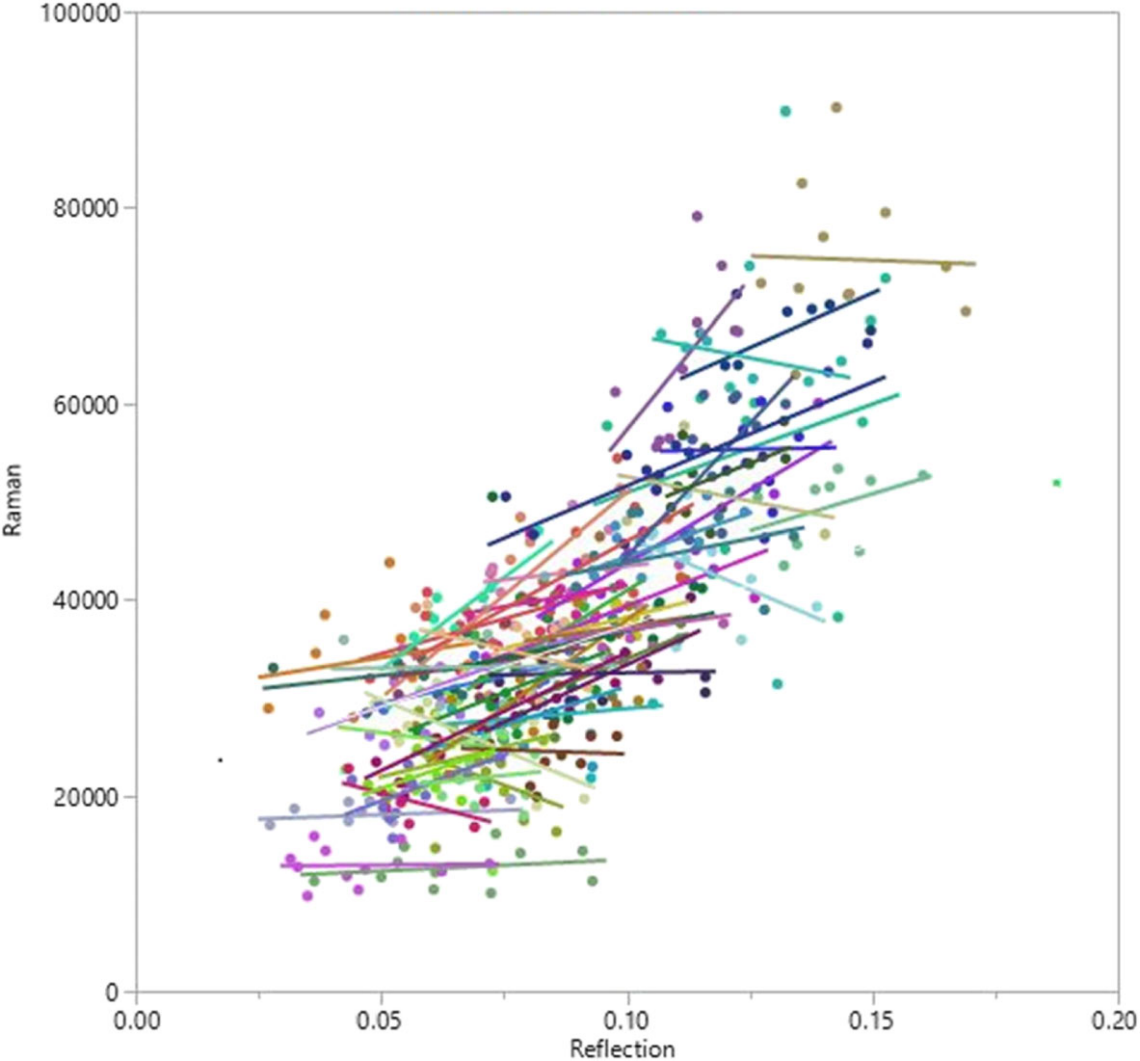
RS compared with RRS intensities for 52 women over 12 time points. Colored lines represent the slope for each person over the year. Per maximum likelihood, the overall between-person correlation between the two skin carotenoid scores was 0.76 (*P* < 0.001) and the overall within-person correlation coefficient was 0.30 (*P* < 0.03). RS, reflection spectroscopy; RRS, resonance Raman spectroscopy

Finally, we examined the relationship of optically assessed SCS to self-reported dietary intake and to total plasma carotenoid concentrations across 1 year. To assess the correlation between plasma and skin carotenoids, we used the skin carotenoid measurements from the same day that blood was drawn (4 time points; once each season). For the correlation of plasma and dietary carotenoids, we used the closest dietary recall preceding the blood draw. To assess correlation between SCS and dietary carotenoids, we used the closest preceding dietary recall at each month (12 time points; once each month). Maximum likelihood estimation was used to calculate the overall correlation of each pair of variables of interest using all available measurements. The method used [32] provides estimates of both the within-individual correlation and the between-individual correlation across the course of the study and correctly accounts for the multiple measurements on each subject. The relationship between RRS and RS was further investigated by fitting a mixed effects linear model (Fig. 1). In the model used, referred to as a random slopes model, the overall slope was modeled as a fixed effect while allowing for a random deviation from the overall slope for each individual. The correlation between observations arising from each individual was also accounted for in the model, and BMI was included as a covariate. All analyses were conducted using SAS version 9.4; (SAS Institute, Inc., Cary, North Carolina) and a *P* value of < 0.05 was considered significant. As adapted from Mukaka for studies related to medical research [33], the interpretation of the correlation coefficients was as follows: 0.00–0.49 = weak, 0.50–0.69 = moderate, and 0.70+ = strong.

## Results

### Seasonal differences in study variables

There were no seasonal differences in RS intensities (*P* = 0.500); however, RRS scores were marginally lower in summer than in spring or fall (*P* < 0.001) (Table 1). Total plasma carotenoid concentrations also differed marginally by season (*P* = 0.025), with the lowest occurring in summer and the highest occurring in spring. Neither self-reported VF (*P* = 0.441) nor self-reported carotenoid intake (*P* = 0.983) differed by season.

**Table 1:** Mean (± SEM) values of study variables by season among US women (*N* = 52) living in North Dakota, USA

	Spring	Summer	Fall	Winter	*P*-value
	Mean ± SEM				
Skin carotenoid status					
RRS intensities^b^	38,238^x^ ± 2617	34,629^y^ ± 1910	37,044^x^ ± 2043	36,563^xy^ ± 1818	< 0.001
RS intensities^b^	0.091 ± 0.004	0.087 ± 0.004	0.088 ± 0.004	0.088 ± 0.004	0.500
Dietary Intake^a^					
Vegetable and fruit (g/d)	188 ± 16	220 ± 16	194 ± 16	187 ± 18	0.441
Dietary carotenoids (mg/d)	12.1 ± 0.76	12.0 ± 0.97	11.9 ± 0.72	11.9 ± 1.11	0.983
	Geometric mean -1SEM, +1SEM				
Total plasma carotenoids (μM/L)^c^	2.53; 2.36, 2.71 ^x^	2.26; 2.11, 2.42 ^y^	2.46; 2.30, 2.64 ^xy^	2.34; 2.18, 2.51 ^xy^	0.025

^a^Measured by the Automated Self-Administered 24-Hour (ASA24) Dietary Assessment Tool at 36 times over the year

^b^Measured at 12 times over the year

^c^Measured at 4 times over the year

•Analyzed carotenoids were α- and β-carotene, β-cryptoxanthin, lycopene, lutein and zeaxanthin.

x,y, Values between seasons with the same superscript letters are not significantly different (*P* > 0.05) by Tukey contrasts.

RS, reflection spectroscopy.

RRS, resonance Raman spectroscopy.

### Correlations between RS and RRS

At baseline, RS and RRS were strongly correlated (*r* = 0.86; *P* < 0.001). Figure 1 displays the relationship between RS and RRS over the year. Based on the mixed linear model fit of the data, the between-person correlation was strong (*r* = 0.76; *P* < 0.001), but the overall within-person correlation was weak (*r* = 0.30; *P* = 0.03). Each individual’s slope is displayed and within-person correlations ranged from *r* = − 0.71 to 0.87; median: *r* = 0.30.

### Baseline and full-year pairwise correlations

Baseline and between-individual correlations across the year are presented in Table 2. RS was strongly correlated with plasma carotenoids at baseline (*r* = 0.70; *P* < 0.001), but moderately across the year (*r* = 0.65; *P* < 0.001), as was RRS (*r* = 0.77 and 0.69, respectively; *P* < 0.001). At baseline, self-reported VF was weakly correlated with RRS (*r* = 0.33; *P* = 0.016) but not with RS (*P* = 0.053) or plasma carotenoids (*P* = 0.050). Across the year, self-reported VF intake was weakly correlated with RS and RRS (*r* = 0.37 for both; *P* < 0.01), and with plasma carotenoids (*r* = 0.36; *P* = 0.008). Dietary carotenoids were not correlated with RS (*P* = 0.500), RRS (*P* = 0.057), or plasma (*P* = 0.089) at baseline, but were moderately correlated with VF intake (*r* = 0.56; *P* < 0.001). Over the year, dietary carotenoids were weakly correlated with RRS (*r* = 0.29; *P* = 0.034) and plasma carotenoids (*r* = 0.34; *P* = 0.15), and moderately correlated with VF intake (*r* = 0.51; *P* < 0.001).

**Table 2:** Correlation coefficients^a^ and 95% confidence intervals of skin carotenoids, plasma carotenoids, and self-reported vegetable and fruit intake at baseline and across one year

	Total plasma carotenoids (μM/L)^d^		VF (cup eq.)^b^		Dietary carotenoids (mg/d)^b^	
	Baseline	Year	Baseline	Year	Baseline	Year
Reflection spectroscopy^c^	0.70*** (0.53, 0.82)	0.65*** (0.46, 0.79)	0.27 (0.00, 0.51)	0.37** (0.10, 0.58)	0.10 (−0.18, 0.36)	0.24 (− 0.03, 0.48)
Resonance Raman spectroscopy^c^	0.77*** (0.63, 0.86)	0.69*** (0.52, 0.81)	0.33* (0.06, 0.55)	0.37** (0.11, 0.59)	0.27 (0.00, 0.51)	0.29* (0.02, 0.52)
Total plasma carotenoids (μM/L)^d^			0.27 (0.00, 0.51)	0.36** (0.10, 0.58)	0.24 (−0.03, 0.48)	0.33* (0.06, 0.55)
VF (cup eq.)^b^					0.56*** (0.34, 0.72)	0.51*** (0.27, 0.69)

^a^Pairwise correlations at baseline were estimated using Pearson coefficients and full-year between-individual correlations were estimated using a mixed model

^b^Measured by the Automated Self-Administered 24-Hour (ASA24) Dietary Assessment Tool at 36 times over the year

^c^Measured at 12 times over the year

^d^Measured at 4 times over the year

Analyzed carotenoids were α- and β-carotene, β-cryptoxanthin, lycopene, lutein and zeaxanthin

*Significant at *P* < 0.05

**Significant at *P* < 0.01

***Significant at *P* < 0.001

## Discussion

This study demonstrated that SCS measured by RS is highly correlated with RRS and with plasma carotenoid concentrations and is weakly correlated with dietary intake of carotenoids or VF measured by self-report. If plasma carotenoid concentrations are considered the reference biomarker, then our results indicate that SCS may be considered a superior method to self-report for assessing VF intake. However, multiple assessment methods may be needed to gain a more complete assessment of VF intake. Self-report methods can be expensive if interviewer-assisted, and are potentially subject to participant bias such as social desirability [34], whereas SCS cannot detect intake of non-carotenoid-rich VF, such as potatoes or mushrooms. Self-report, by either 24-HR or food frequency questionnaire (FFQ), is the only way to determine overall dietary patterns. Similar to blood carotenoids, SCS is affected by factors that influence the absorption, transport, and metabolism of carotenoids [35], such as the food matrix [36] or genetics [37], and may be affected by environmental factors, such as smoking, sun exposure, or adiposity, therefore it is (thus far) not possible to link a given SCS score with a specific intake of VF. Regardless, SCS is another tool that public health researchers and clinicians may use for surveillance and assessment of the effectiveness of interventions. For example, SCS is being used to determine the effectiveness of a cost-offset Community Supported Agriculture (CSA) project among low-income children and their caregivers [38] and to determine the effectiveness of a healthy corner store initiative in North Carolina [20, 39]. The present study is especially pertinent as it is the first longitudinal study to assess the relative validity of RS, which is more widely available compared to the RRS technology.

Many factors may have influenced our observed results. Scarmo and colleagues compared SCS (measured by RRS) values over a 6-month period and found good agreement over time, although they also found that RRS scores were lower in summer, as we found [14]. We did not find strong correlations between SCS and dietary carotenoids in the current study. However, Mayne et al., compared SCS (measured by RRS) to dietary carotenoids measured by a 2-month FFQ (representing “usual” intake) and found a correlation of 0.57 [10]. To put this in perspective, many FFQs are validated compared to blood carotenoid concentrations, with correlations ~ 0.2–0.5 [40–42]. As a year’s worth of 24-HR may also be considered to approximate “usual” intake, we found similar correlations with VF intake of *r* = 0.37 using either SCS method for between-person correlations; generally the only coefficient measured in validation studies. However, within an individual, we found very poor correlations with plasma and dietary intakes for both SCS methods (data not shown), suggesting that SCS is a better indicator of group intakes than of individual intakes. This may be reflective of a number of factors: 1) As there was only marginal variation between seasons in either self-reported VF or dietary carotenoids, these low levels of variability in intake of VF and dietary carotenoids may be at least partially responsible for the low observed correlations; 2) vegetables are often consumed in mixed dishes, which are notoriously difficult to assess and rely upon the best judgement of the respondent to identify a food code similar to what they consumed, which is linked to a recipe similar to that consumed by the respondent, which is then linked to nutrient databases that have their own set of errors. Factors other than vegetable and fruit intake affect blood carotenoid concentrations, and therefore may be expected to impact observed SCS as well. Animal foods, such as eggs (a rich source of lutein), shellfish and salmon contain carotenoids. Genetics is probably the most influential; single nucleotide polymorphisms in the β, β carotene 15,15′-oxygenase 1 [43, 44] gene, as well as others [45–47], are beginning to be described as affecting blood carotenoid concentrations. Other factors may also play a role, such as smoking, adiposity, sun exposure and oxidative stress exposure, such as chronic illness [15]. Individuals with metabolic syndrome have lower levels of SCS than healthy individuals [48]. In addition, carotenoids are sequestered into other body tissues, such as adipose [49], eye [50], bone [51], and brain [52], leading to competition for skin deposition with resultant variability in tissue levels of carotenoids [53–55]. The kinetics of carotenoids depositing into skin are still not well-defined, and it is unknown how SCS responds to different doses of VF.

SCS as a biomarker of VF intake has been proposed and several validation studies have been published [9]. However, this is the first study to compare RS to RRS, and the first to compare SCS to 24-HR data. Specifically, strengths of this study include the full year longitudinal nature of the data as it allowed us to examine correlations both between and within individuals over time, as well as cross-sectionally at baseline. The use of the ASA24 can also be considered a strength as it collects detailed dietary information. This study also has several limitations to consider. The sample is small, homogeneous, and not generalizable to the larger US population. The women who participated were also overweight; only 23% had obesity. Thus, our results may differ in lean populations and people with obesity. SCS, in addition to the caveats listed above, is limited in that it only responds to carotenoid-rich foods. However, it is an objective measure, and current dietary guidance specifically recommends intake of dark green and orange and red vegetables, which are rich in carotenoids [56]. SCS was poorly correlated with baseline self-reported VF intake, which is not surprising considering that 24-HRs estimate only short-term intake, while SCS may be expected to represent longer-term tissue stores. The RS device used in this study was a prototype, and the commercially-available RS device may be more sensitive.

## Conclusions

In conclusion, we found that SCS is strongly correlated to plasma carotenoids, but weakly to self-reported VF intake. This begs the question, which method do we choose to evaluate our interventions? In this long-term study, we have shown that plasma carotenoid concentrations, the gold standard for VF intake, are strongly correlated with RS and RRS but only weakly correlated with self-reported dietary intake of VF. SCS, particularly as measured by RS, is rapid, non-invasive and does not require specialized training or data processing compared to blood carotenoid analysis. Our results indicate that SCS assessed via RRS and RS is more strongly associated with plasma carotenoids than is self-reported VF or carotenoid intake. At this point, there are a variety of methods to assess changes in VF intake, and we cautiously suggest that SCS can be another tool that researchers and clinicians may utilize when attempting to change dietary behavior or during population-level monitoring and surveillance.

## Data Availability

Data described in the manuscript, code book, and analytic code will be made available upon request pending approval and signed agreements.

## Abbreviations

ASA24: Automated Self-Administered 24-Hour Dietary Assessment Tool
RRS: resonance Raman spectroscopy
RS: reflection spectroscopy
SCS: skin carotenoid status
VF: vegetables and fruits
